# Supplementary material for: Sfrp3 modulates stromal–epithelial crosstalk during mammary gland development by regulating Wnt levels
Source: Nat Commun. 2019 Jun 6;10:2481. doi: 10.1038/s41467-019-10509-1 (PMC6554275; doi:10.1038/s41467-019-10509-1)
Supplement: Supplementary file 1 — Supplementary Information [file 41467_2019_10509_MOESM1_ESM.pdf]

Sfrp3 modulates stromal–epithelial crosstalk during mammary gland development by regulating Wnt levels

Bernascone et al.

Supplemental figures with corresponding titles and legends and supplemental tables with corresponding titles and legends (non-Excel)

Supplementary Figures

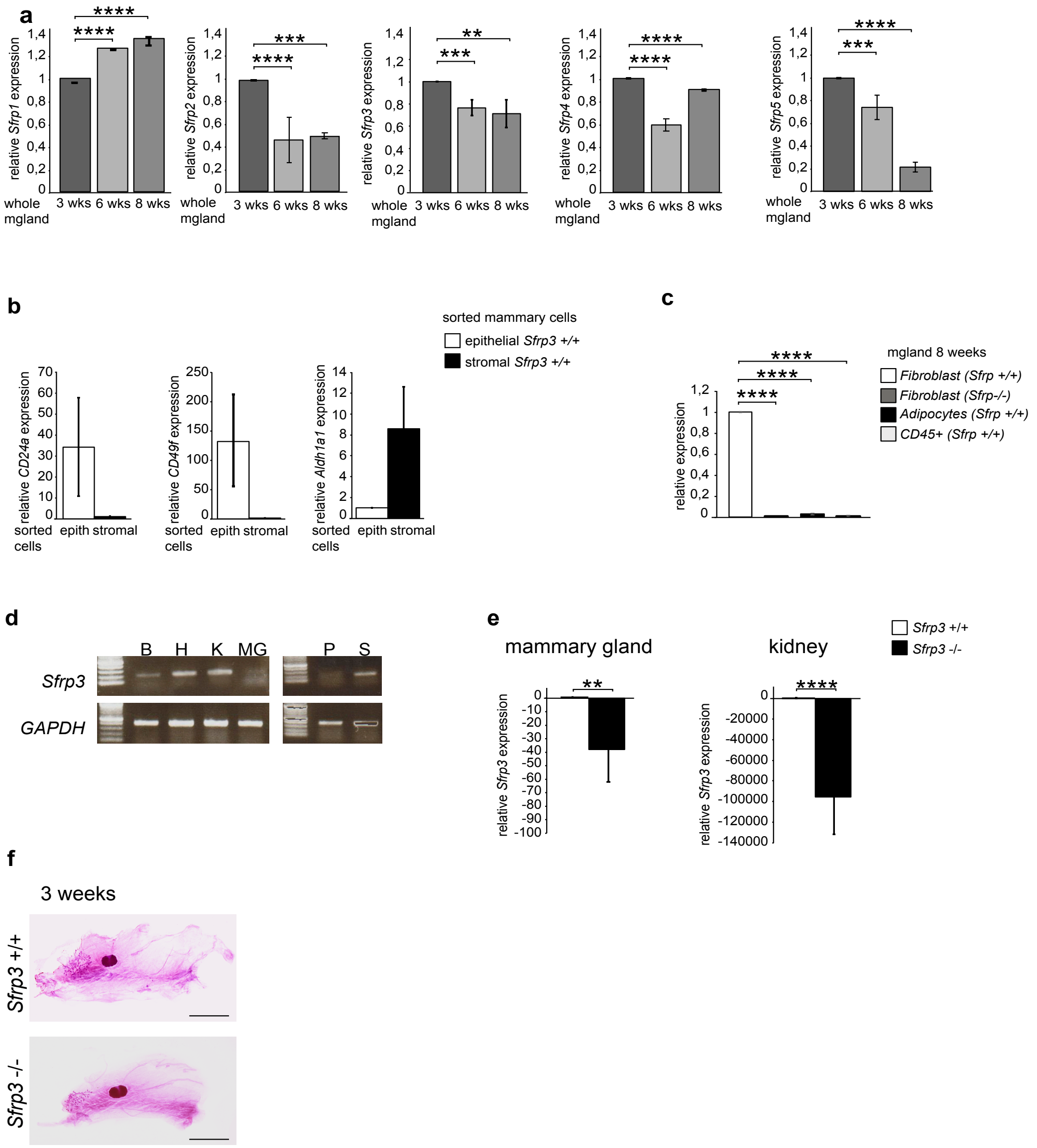

Supplementary fig. 1. Stromal and epithelial cells of the MG express Sfrps

(a) Relative mRNA level of *Sfrp1*, *Sfrp2, Srfp3, Sfrp4,* and *Sfrp5* in whole mammary gland. n = 3 pools, each pool collected from 15 females at 12 weeks of age. (b) Relative mRNA level of *CD24a* (left), *CD49f* (middle) and *Aldhala1* (right) in FACS-sorted wildtype mammary epithelial and stromal cells. n = 2 pools, each pool collected from 15 females at 12 weeks of age. The expression level of *CD24a*, *CD49f* and *Aldhala1* is used as control of purity of sorted cells populations. (c) RT-PCR showing expression of *Sfrp3* in isolated cell populations of fibroblast *Sfrp3*$^{+/+}$ and *Sfrp3*$^{-/-}$, adipocites *Sfrp3*$^{-/-}$, and CD45+ cells *Sfrp3*$^{-/-}$. n = 3 pools, each pool collected from mammary glands of 15 females at 8 weeks of age. (d) RT-PCR showing expression of *Sfrp3* and *GAPDH* (as normalizer gene) in tissues from adult *Sfrp3*$^{+/+}$ mice. B, brain; H, heart; K, kidney; MG, mammary gland; P, pancreas; S, spleen. Image is representative of 3 RT-PCR analysis performed (e) Relative *Sfrp3* mRNA level in mammary gland and kidney of *Sfrp3*$^{+/+}$ and *Sfrp3*$^{-/-}$ mice at 10 weeks of age. n = 6 females. Data represent mean ± SD (** $p < 0.01$, **** $p < 0.001$). (f) Representative images of at least 3 sections of whole mount of mammary glands from *Sfrp3*$^{+/+}$ and *Sfrp3*$^{-/-}$ virgin females at 3 weeks of age (n = 4). Scale bar: 0.5 cm. Source data are provided as a Source Data file.

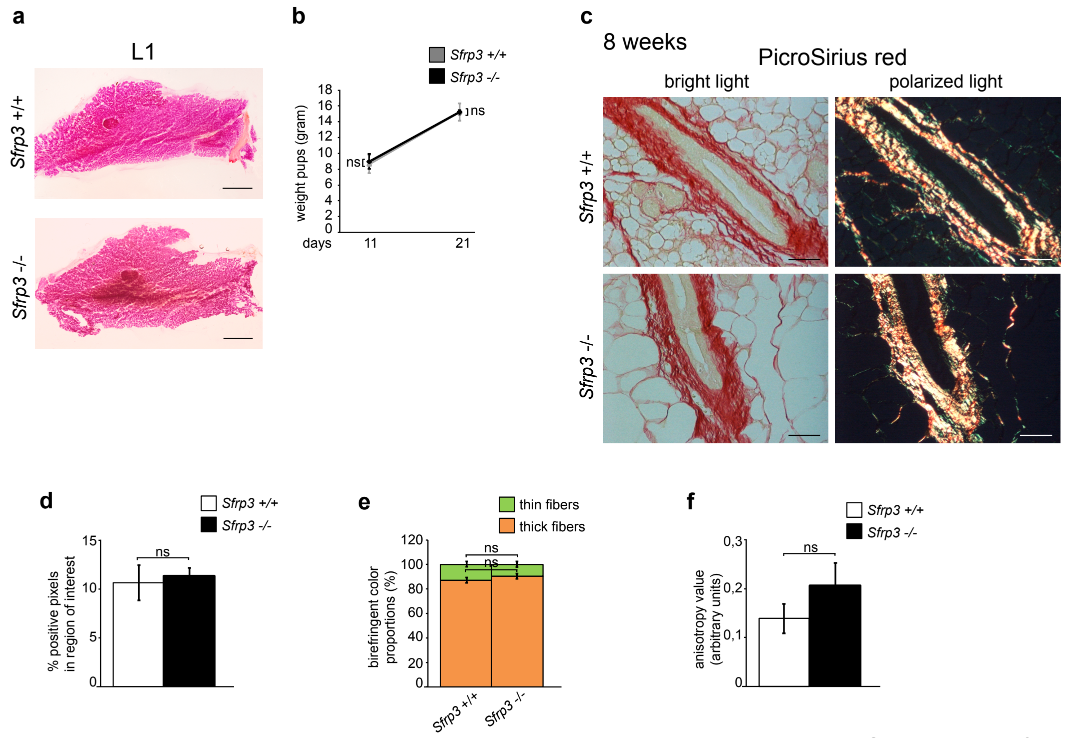

Supplementary fig.2. *Sfrp3* loss does not affect pregnancy or stromal collagen.

(a) Representative whole mount of mammary glands from *Sfrp3*$^{+/+}$ and *Sfrp3*$^{-/-}$ females at lactation day 1 (L1). Representative images whole mount of MG of n = 4. Scale bar: 0.5 cm. (b) Weight of *Sfrp3*$^{+/+}$ and *Sfrp3*$^{-/-}$ pups at day 11 and 21 of weaning. n = 7. Data represent mean ± SD (ns, not significant).

(c) Representative images of mammary sections stained with PicroSirius red taken with bright (left) and polarized light (right) from *Sfrp3*$^{+/+}$ and *Sfrp3*$^{-/-}$ females at 8 weeks of age. Scale bar: 50 µm. (D-F) Quantification of collagen content (d), thick and thin fibers (e), and anisotropy level of collagen (f) in *Sfrp3*$^{+/+}$ and *Sfrp3*$^{-/-}$ mammary glands. n = 3. For (d-f) data represent mean ± SD (ns, not significant). Source data are provided as a Source Data file.

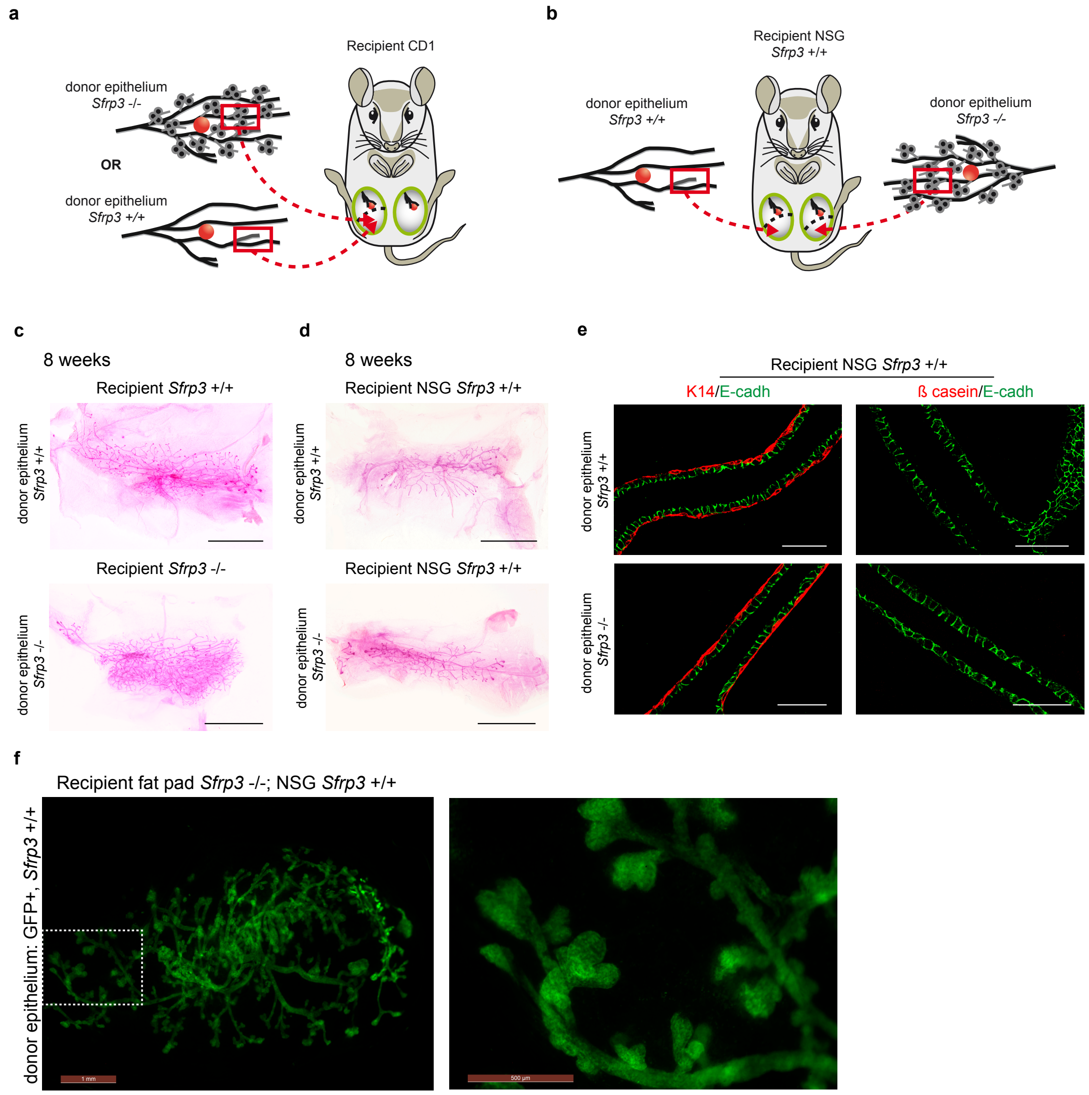

Supplementary fig.3 *Sfrp3*$^{+/+}$ stroma does not affect ductal branching or structure

(a) The strategy of the mammary epithelial transplantation in CD1 females. Both #4 glands of the recipient mouse are shown together with the rudimentary epithelial tree and the lymph node. The dashed line in the right gland shows the location of the cut for clearing fat pad. The left gland is not operated and allowed to develop. The arrows illustrate the origin of donor tissues (either *Sfrp3*$^{+/+}$ or *Sfrp3*$^{-/-}$ epithelium) and the location to which they are transplanted. (b) The strategy of the mammary epithelial transplantation in immunocompromised (NSG) females. It is identical to (a) except both #4 glands of the recipient mouse are transplanted, the right with *Sfrp3*$^{+/+}$ epithelium and the left with *Sfrp3*$^{-/-}$ epithelium. (c) Whole mount of transplanted mammary glands of CD1 females analysed 5 weeks after the operation. Upper panel, *Sfrp3*$^{+/+}$ epithelium is transplanted in *Sfrp3*$^{+/+}$ stroma (n = 7); bottom panel, *Sfrp3*$^{-/-}$ epithelium is transplanted in *Sfrp3*$^{-/-}$ stroma (n = 2). Scale bar: 0.5 cm. (d) Whole mount of transplanted mammary glands of immunocompromised (NSG) females analysed 5 weeks after the operation. *Sfrp3*$^{+/+}$ epithelium (upper, n = 5) and *Sfrp3*$^{-/-}$ epithelium (bottom n = 3) are transplanted in *Sfrp3*$^{+/+}$ stroma. Scale bar: 0.5 cm. (e) Immunofluorescence staining for K14 and E-cadherin (left) and ß casein and E-cadherin (right) on mammary sections from NSG females transplanted with *Sfrp3*$^{+/+}$ (upper panel) and *Sfrp3*$^{-/-}$ (bottom panel) epithelium. Scale bar: 50 µm. Images are representative of at least 10 MG sections analysed. (f)

Immunofluorescence staining for GFP-marked epithelial cells on whole mammary sections from NSG females transplanted with GFP+; *Sfrp3*$^{+/+}$ donor epithelium into *Sfrp3*$^{-/-}$ fat pad. Right image is the magnification of squared region in the left panel. Representative image of n=3. Scale bar: 1 mm.

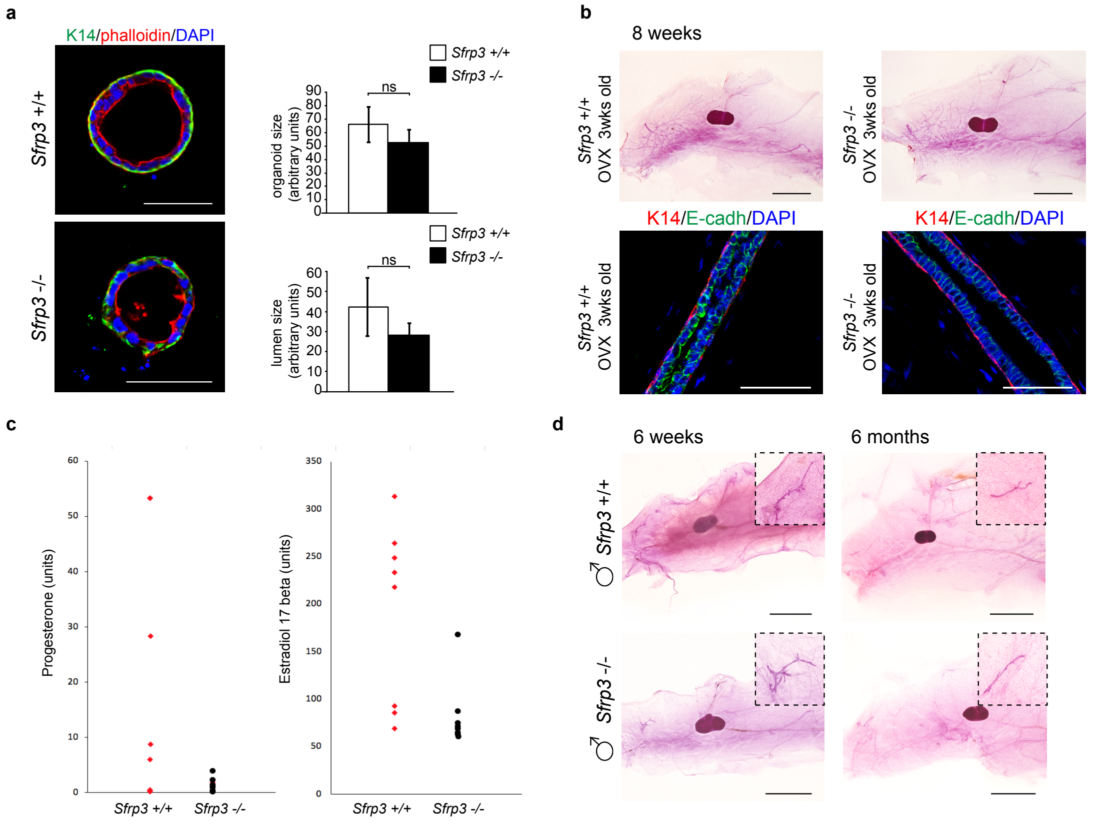

Supplementary fig.4 Branching in *Sfrp3*$^{-/-}$ MG is not due to loss of *Sfrp3* in the epithelium and it is under hormonal control.

(a) Left, images of *Sfrp3*$^{+/+}$ and *Sfrp3*$^{-/-}$ mammary organoids immunostained for K14, actin (phalloidin) and DAPI, after 6 days of culture. Scale bar: 50 µm. Right, quantification of organoid size (upper graph) and lumen size (bottom graph) in *Sfrp3*$^{+/+}$ and *Sfrp3*$^{-/-}$ mammary organoids after 6 days of culture. n = 63 *Sfrp3*$^{+/+}$ organoids (collected from 5 females); 47 *Sfrp3*$^{-/-}$ organoids (collected from 4 females). Data represent mean ± SD (ns, not significant). (b) Upper panel, whole mount of mammary glands from *Sfrp3*$^{+/+}$ and *Sfrp3*$^{-/-}$ females ovariectomized at 3 weeks of age and analysed 5 weeks after. Scale bar: 0.5 cm. Lower panel, immunofluorescence staining for K14, E-cadherin and DAPI on mammary sections from ovariectomized *Sfrp3*$^{+/+}$ and *Sfrp3*$^{-/-}$ females. Scale bar: 50 µm. Images are representative of at least 4 MG sections analysed (n=3). (c) Quantification of serum levels of estrogen and progesterone of *Sfrp3*$^{+/+}$ and *Sfrp3*$^{-/-}$ mice at 8 weeks of age. n = 8 females. Data represent mean ± SD (Progesterone, p = 0.0519, Estrogen, p =0.0088). (d) Representative whole mount of mammary glands of *Sfrp3*$^{+/+}$ and *Sfrp3*$^{-/-}$ males analysed at 6 weeks and 6 months of age. Images are representative of 4 MG sections analysed. Scale bar: 0.5 cm. The bracketed area shows the rudimentary epithelial tree at higher magnification. Source data are provided as a Source Data file.

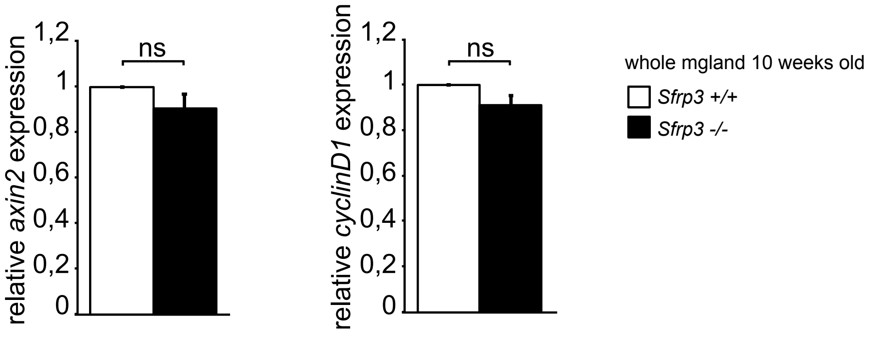

Supplementary fig.5 ***Sfrp3$^{-/-}$* *axin2*** **and** ***cyclinD1*** **levels in whole MG.**

Relative mRNA level of *axin2* (left) and *cyclinD1* (right) in whole mammary gland of *Sfrp3*$^{+/+}$ (n = 2) and *Sfrp3*$^{-/-}$ (n = 4) mice at 10 weeks of age. For data represent mean ± SD (** p < 0.01, *** p < 0.001, **** p < 0.001, ns, not significant). Source data are provided as a Source Data file.

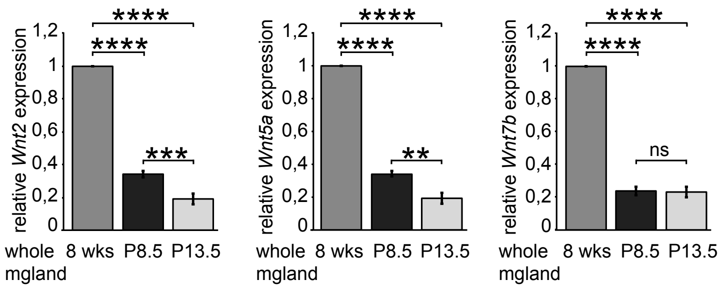

Supplementary fig.6

Expression profile of ***Wnt2*****,** ***Wnt5a*** **and** ***Wnt7b*** **in adult and pregnant female mice.**

Relative mRNA level of *Wnt2* (left), *Wnt5a* (middle) and *Wnt7b* (right) of whole mammary gland at 8 weeks of age (n = 4) and at pregnancy days P8.5 (n = 3) and P13.5 (n = 4). For data represent mean ± SD (** p < 0.01, *** p < 0.001, **** p < 0.001, ns, not significant). Source data are provided as a Source Data file.

Supplementary Table 1. Functional annotation of 102 DEGs between *Sfrp3*$^{+/+}$ and *Sfrp3*$^{-/-}$ epithelial cells.

Gene Ontology (GO) enrichment of biological process (BP)

CATEGORY	GO ID	TERM	COUNT	FDR
BP	GO: 0009888	tissue development	41	5,20E-12
BP	GO: 0009887	organ morphogenesis	24	7,7E-6
BP	GO: 0002009	morphogenesis of an epithelium	18	6,57E-6
BP	GO: 0048513	animal organ development	46	2,94E-6
BP	GO: 0048729	tissue morphogenesis	20	2,35E-6

Gene Ontology (GO) enrichment of biological process (BP). The top five GO BP terms with a false discovery rate (FDR) $\leq$ 0.001 % are shown.

David Functional Annotation

ANNOTATION CLUSTER	REPRESENTATIVE ANNOTATION TERMS	COUNT	ENRICHMENT SCORE
1	cellular response to chemical stimulus; negative regulation of apoptotic process; positive regulation of phosporylation; positive regulation of protein metabolic process; protein phosphorylation; regulation of cell proliferation; regulation of cellular protein metabolic process	63	4,9
2	insulin-like growth factor binding; regulation of cell growth	33	4,6
3	mammary gland alveolus development	8	4,1

David Functional Annotation. The top 3 enriched clusters are shown.
