# Supplementary material for: Sfrp3 modulates stromal–epithelial crosstalk during mammary gland development by regulating Wnt levels
Source: Nat Commun. 2019 Jun 6;10:2481. doi: 10.1038/s41467-019-10509-1 (PMC6554275; doi:10.1038/s41467-019-10509-1)
Supplement: Supplementary file 2 — Description of Additional Supplementary Files [file 41467_2019_10509_MOESM2_ESM.doc]

**Title: Supplementary Data 1. NGS analysis in *Sfrp3*^-/-^ compared to *Sfrp3*^+/+^ epithelial cells.
Description:** Related to Figure 7; NGS analysis

**Title: Supplementary Data 2. 50 top down-regulated genes in *Sfrp3*^-/-^ compared to *Sfrp3*^+/+^** **epithelial cells.
Description:** List of 50 top (log2 fold change < -2) downregulated genes in FACS-sorted *Sfrp3*^-/-^ epithelial cells compared to *Sfrp3*^+/+^ epithelial cells.

**Title: Supplementary Data 3. 50 top up-regulated genes in *Sfrp3*^-/-^ compared to *Sfrp3*^+/+^** **epithelial cells.
Description:** List of 50 top (log2 fold change > 2) upregulated genes in FACS-sorted *Sfrp3*^-/-^ epithelial cells compared to *Sfrp3*^+/+^ epithelial cells.

**Title: Supplementary Data 4. Real-Time qPCR primers
Description:** Related to STAR METHOD; Primers for quantitative Real-Time PCR.
