# Supplementary material for: Sfrp3 modulates stromal–epithelial crosstalk during mammary gland development by regulating Wnt levels
Source: Nat Commun. 2019 Jun 6;10:2481. doi: 10.1038/s41467-019-10509-1 (PMC6554275; doi:10.1038/s41467-019-10509-1)
Supplement: Supplementary file 8 — Source Data [file 41467_2019_10509_MOESM8_ESM.zip › Source data/Source data blots Fig. 6.pdf]

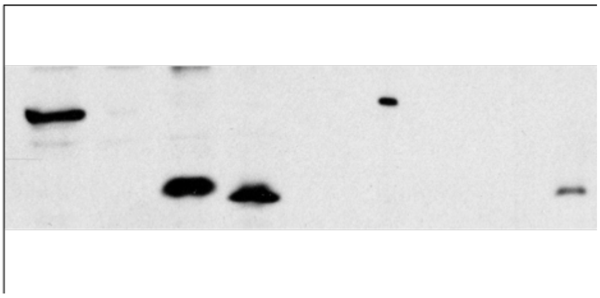

Figure 6f bottom panel
Blotted with anti myc 1:1000

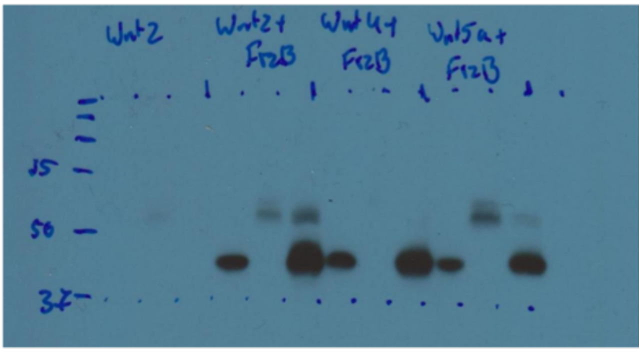

Figure 6g top panels
Blotted with anti myc 1:1000

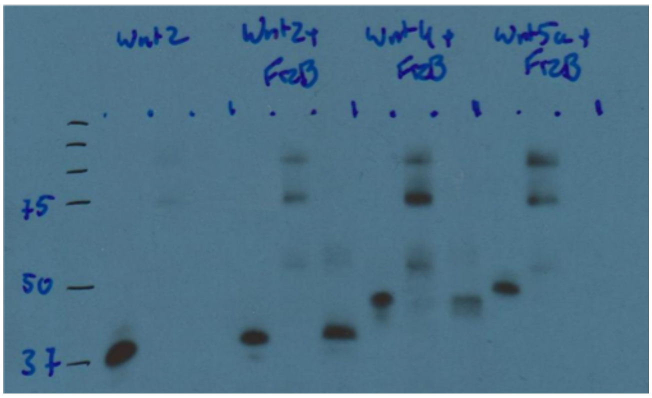

Figure 6g top panels
Blotted with anti V5 1:500

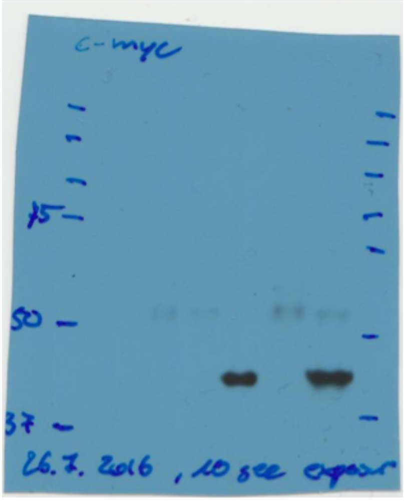

Figure 6g bottom panels
Blotted with anti myc 1:1000

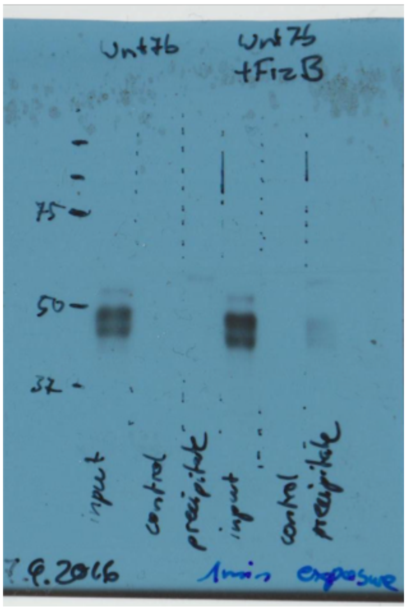

Figure 6g bottom panels
Blotted with anti HA 1:250

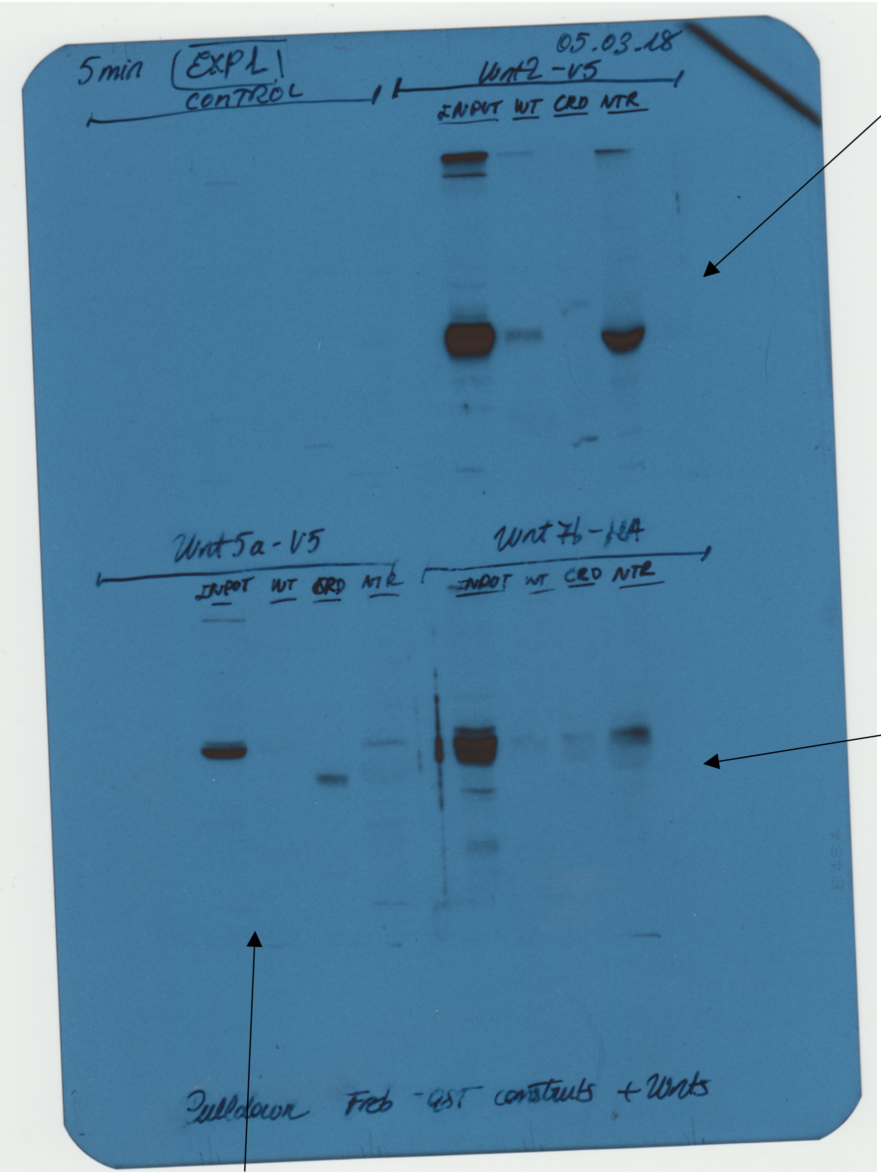

Fig. 6h top panels Blotted with anti V5 1:500

Fig. 6h-bottom panels Blotted with anti HA 1:250

Fig. 6h-top panels Blotted with anti V5 1:500
